# Cyclist Effort Features: A Novel Technique for Image Texture Characterization Applied to Larynx Cancer Classification in Contact Endoscopy—Narrow Band Imaging

**DOI:** 10.3390/diagnostics11030432

**Published:** 2021-03-03

**Authors:** Nazila Esmaeili, Axel Boese, Nikolaos Davaris, Christoph Arens, Nassir Navab, Michael Friebe, Alfredo Illanes

**Affiliations:** 1 INKA—Innovation Laboratory for Image Guided Therapy, Otto-von-Guericke University Magdeburg, 39120 Magdeburg, Germany; axel.boese@med.ovgu.de (A.B.); michael.friebe@ovgu.de (M.F.); alfredo.illanes@med.ovgu.de (A.I.); 2 Chair for Computer Aided Medical Procedures and Augmented Reality, Technical University Munich, 85748 Munich, Germany; navab@cs.tum.edu; 3 Department of Otorhinolaryngology, Head and Neck Surgery, Magdeburg University Hospital, 39120 Magdeburg, Germany; nikolaos.davaris@med.ovgu.de (N.D.); christoph.arens@med.ovgu.de (C.A.); 4 IDTM GmbH, 45657 Recklinghausen, Germany

**Keywords:** texture feature extraction, classification, contact endoscopy, narrow band imaging, larynx

## Abstract

Background: Feature extraction is an essential part of a Computer-Aided Diagnosis (CAD) system. It is usually preceded by a pre-processing step and followed by image classification. Usually, a large number of features is needed to end up with the desired classification results. In this work, we propose a novel approach for texture feature extraction. This method was tested on larynx Contact Endoscopy (CE)—Narrow Band Imaging (NBI) image classification to provide more objective information for otolaryngologists regarding the stage of the laryngeal cancer. Methods: The main idea of the proposed methods is to represent an image as a hilly surface, where different paths can be identified between a starting and an ending point. Each of these paths can be thought of as a Tour de France stage profile where a cyclist needs to perform a specific effort to arrive at the finish line. Several paths can be generated in an image where different cyclists produce an average cyclist effort representing important textural characteristics of the image. Energy and power as two Cyclist Effort Features (CyEfF) were extracted using this concept. The performance of the proposed features was evaluated for the classification of 2701 CE-NBI images into benign and malignant lesions using four supervised classifiers and subsequently compared with the performance of 24 Geometrical Features (GF) and 13 Entropy Features (EF). Results: The CyEfF features showed maximum classification accuracy of 0.882 and improved the GF classification accuracy by 3 to 12 percent. Moreover, CyEfF features were ranked as the top 10 features along with some features from GF set in two feature ranking methods. Conclusion: The results prove that CyEfF with only two features can describe the textural characterization of CE-NBI images and can be part of the CAD system in combination with GF for laryngeal cancer diagnosis.

## 1. Introduction

Medical images contain crucial information that is analyzed by clinicians to find abnormalities and diagnose diseases. The level of tortuosity of anatomical structures such as blood vessels is one type of information that can be useful for clinicians. Vascular networks in tumors are irregular in size, shape, and branching pattern, lack the normal hierarchy, and do not display the recognizable features of arterioles, capillaries, or venules [1]. For example, in ophthalmology, retinal vascular tortuosity can be a potential indicator of diseases such as hypertension, diabetes, or atherosclerosis [2]. The changes in the organization and structure of the larynx vocal fold’s blood vessels are directly related to the development of benign and subsequent malignant laryngeal lesions. The manual assessment of vascular structures can, however, result in significant inter-observer variability and with that in subjective diagnosis [3,4].

Nowadays, Computer-Aided Diagnosis (CAD) systems use different feature extraction methods in combination with classification algorithms to assist clinicians in solving such problems. Features extraction is the process of generating features such as color, shape, and texture to describe the content of an image [5]. The significance of these features for describing image characteristics are of great importance and essential for the good performance of the CAD. There are several deep learning-based and hand-crafted feature extraction methods for medical image analysis. The deep learning-based approaches include the automatic features extraction and classification that mostly result in a high performance, but the majority of these approaches are computationally expensive to train, need lots of data and are known as the black art [6,7]. In the biomedical field, texture features are often used for characterizing an image using several hand-crafted feature extraction methods [8,9,10,11,12,13,14]. Although these methods have shown good performances for computing features, they have some drawbacks. Usually, a large number of features is needed for the classification, resulting in computationally expensive solutions. Moreover, most of the proposed features in the literature have limited or no meaning for the clinicians [5,15].

In this work, we propose a novel approach for image texture characterization. The main principle of the proposed approach is to consider an image as an irregular relief surface where different paths can be traced between a starting and an ending point. Each path can be thought of as a Tour de France course profile, where a cyclist needs to perform a specific effort to arrive at the finish line. The effort performed by a large number of cyclists following different paths in the hilly relief image can be representative of the image texture. Using this concept, we have extracted two features that we dubbed the Cyclist Effort Features (CyEfF).

The usability of the proposed approach was tested to classify larynx Contact Endoscopy (CE)—Narrow Band Imaging (NBI) images into benign and malignant classes. CE-NBI is an enhanced endoscopic imaging technique that allows a detailed examination of laryngeal mucosa and provides more precise information about the structure of the superficial capillary network and sub-mucosal vessels in comparison to other endoscopic techniques. The visual evaluation of endoscopic images such as CE-NBI, is a subjective process causing difficulty for clinicians to recognize malignant lesions [3,16,17]. Several computer-based diagnosis approaches were applied to laryngeal endoscopic images to overcome this issue and present complementary information about the state of the larynx for clinicians [18]. Recent studies included a Deep Convolutional Neural Network (DCNN) using laryngoscopic images for larynx cancer detection [19], a set of texture-based features and Deep Learning-based descriptors extracted from endoscopic NBI images for laryngeal Squamous Cell Carcinoma (SCC) detection [20], a set of texture-based and first-order statistical features [21] plus an ensemble of Convolution Neural Networks (CNN) with texture and frequency domain based features [22] for larynx cancerous tissue classification using endoscopic NBI images, a set of features combined with supervised Machine Learning techniques for vascular patterns’ assessment in CE-NBI images and laryngeal cancer diagnosis [23,24,25].

With the primary goal of this work to show the significance of the CyEfF for classification purposes, we have compared the proposed features with two other sets, including 24 Geometrical Features (GF) [24] and 13 Entropy Features (EF) [21] that have been proposed in the literature for the larynx endoscopic image classification. The results showed that the classification performance of the two proposed CyEfF is similar to the performance of other feature sets that includes a greater number of features and indicated the significance of the CyEfF set on improving the classification performance of GF.

## 2. Method

### 2.1. Cyclist Effort Features Formulation

**Figure 1:**
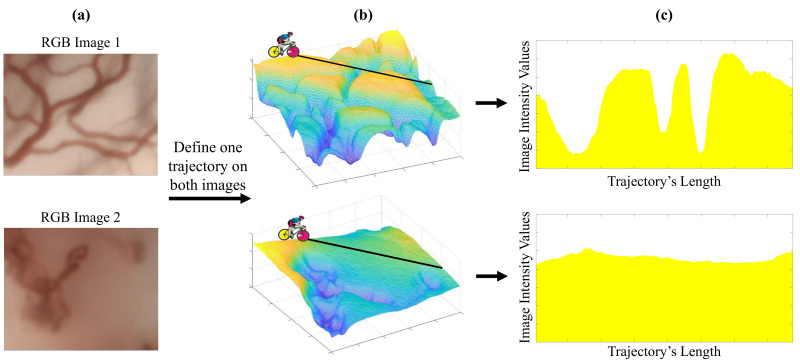
(**a**): RGB 2D image. (**b**): 3D representation of image. (**c**) Stage profile of similar trajectory on two images.

Figure 1 depicts the main idea behind the proposed feature extraction method. We show in Figure 1a two CE-NBI images of vessels. A 2-Dimensional (2D) grayscale image can be viewed as a 3-Dimensional (3D) surface by representing the intensity values of each pixel (being located in the x-y plane) along the z-axis. With that, we can consider each image as a hilly relief surface where a path can be traced between a starting and an ending point (see Figure 1b). We can imagine each of these paths as a Tour de France bicycle race stage. When a cyclist starts racing within an image, one trajectory of cyclist creates a sort of Tour de France stage profile (see Figure 1c). The cyclist needs to make an effort to accomplish each stage. This effort can be assessed by the energy that the cyclist spends and the associated cyclist’s power. We can see in Figure 1c how these two trajectories can involve profiles that require a different degree of effort of a cyclist.

When a large number of cyclists, randomly distributed over the whole image, are performing different trajectories, an average effort of all cyclists can be obtained by computing average energy and power. This average effort can be representative of the image texture. As in the Tour de France, a stage can be classified as flat, mountainy or hilly. Our main idea using this new concept (cyclist energy and power features) is to classify texture in images since the average effort of cyclists in an image can vary according to its characteristic patterns.

There are three primary forces that a cyclist must overcome in order to move forward [26]:

- Gravity Force ($F_{G}$): is one of the critical factors in cycling because a cyclist needs to fight against it cycling uphill. It can be calculated in metric units as $F_{G}=g\cdot \sin\left(\arctan\left(S\right)\right)\cdot m$, where *S* is the percentage grade to measure the steepness of a hill. *g* is the gravitational force constant and *m* is the combined weight of cyclist and bike.
- Rolling Resistance Force ($F_{R}$): is the friction between the tires and the road surface and is calculated in metric units as $F_{R}=g\cdot \cos\left(\arctan\left(S\right)\right)\cdot m\cdot C_{r}$, where $C_{r}$ is a dimensionless parameter that captures the bumpiness of the road and the quality of tires.
- Air Resistance Force ($F_{A}$), which for the purposes of this work can be assumed to be a constant.

The total force resisting the cyclist is, therefore, $F_{T}=F_{G}+F_{R}+F_{A}$ and is the key parameter to calculate the cycling power and energy as:

(1) $$P=F_{T}\cdot v\;\mathrm{and}\;E=F_{T}\cdot v\cdot t$$

where *v* is the cycling velocity and *t* is the time duration of the cyclist’s effort. These two parameters are used to compute the textural features proposed in this work.

### 2.2. Cyclist Effort Features Computation

**Figure 2:**
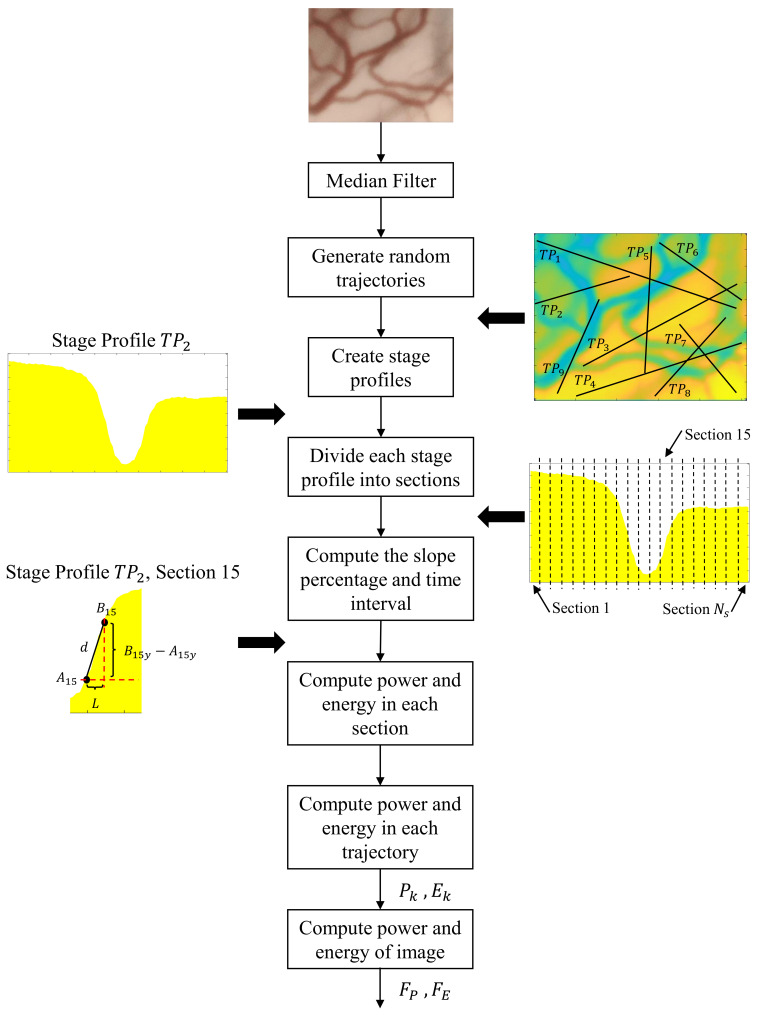
Feature extraction flowchart.

The block diagram in Figure 2 shows the feature extraction process. Since the computation of cyclist power and energy requires the estimation of slopes in an image are known to be sensitive to high-frequency noise, the image is first pre-processed using a Median filter. For this filter, the kernel size was set empirically to 5×5 after visually evaluating the effect of three different kernel sizes on some randomly selected CE-NBI images. Then, different straight-line trajectories are generated inside the image between randomly selected starting and ending coordinate points. The trajectories need to include sufficient data from the image; hence each trajectory had at least 50-pixels length, equivalent to around 1% of the image’s size. The pixel intensity values under each trajectory line are stored as vector arrays that correspond to race profiles.

Let $TP_{k}\left(i\right)$ be the pixel value of the trajectory profile vector *k* (with $k=1,...,N_{k}$ and $N_{k}$ corresponding to the total number of trajectories generated in an image) at the pixel index *i* ($i=1,...,N_{i}$ with $N_{i}>50$ being the length of the vector $TP_{k}$). For computing the cycling power and the cycling energy features of a full image, the power and energy of these individual $TP_{k}$ trajectories should be first calculated. For that, each trajectory vector $TP_{k}$ is first divided into $N_{s}$ non-overlapped sections of length *L*. Then the power and energy of each one of these sections are computed using Equation (1). Figure 2 shows an example of the calculation process for the section $N_{s}=15$. The section’s slope percentage $S_{n}$ and time interval $t_{n}$ have to be estimated for each generated section *n* ($n=1,...,N_{s}$). A trajectory can be seen as a curve in the 2-D plane, where the x-axis correspond to the pixel elements *i* and the y-axis correspond to the value of the vector $TP_{k}\left(i\right)$. Following this representation, let $A_{n}=\left(A_{nx},A_{ny}\right)$ and $B_{n}=\left(B_{nx},B_{ny}\right)$ being the starting and ending coordinate points, respectively, of the trajectory in section *n*. Then, the time interval can be computed as a simple ratio between a distance and the velocity as:

(2) $$t_{n}=\frac{d(A_{n},B_{n})}{v}$$

where $d(A_{n},B_{n})$ corresponds to the Euclidean distance between $A_{n}$ and $B_{n}$ and *v* to the cyclist velocity. The section’s slope percentage $S_{n}$ can be calculated as the ratio between the y-axis jump between $A_{n}$ and $B_{n}$ and the length of the section *n*:

(3) $$S_{n}=\frac{B_{ny}-A_{ny}}{L}$$

Using the estimated $S_{n}$ and $t_{n}$ it is possible to compute the power $P_{n}$ and energy $E_{n}$ of section *n* using the Equation (1). Then the power and energy of a trajectory $TP_{k}$ are computed as:

(4) $$P_{k}=\sum_{n=1}^{N_{s}}P_{n}\;\mathrm{and}\;E_{k}=\sum_{n=1}^{N_{s}}E_{n}$$

$F_{P}$ and $F_{E}$ of the full image are computed as the average values of $P_{k}$ and $E_{k}$, respectively.

The approach was implemented in MATLAB R2019a and executed on a PC with a CPU operating at 1.60 GHz resulting in an average execution time of 0.71 seconds per image.

## 3. Experiments

### 3.1. Data Acquisition and Dataset Generation

The usability of the proposed method was evaluated in CE-NBI image classification. An updated version of the Dataset IV in [24] including 48 patients and 2701 CE-NBI images was used. Patients’ data were anonymized and the biopsy results were used to label images into benign and malignant lesions according to the WHO classification [27]. The benign group involved images of patients with Cyst, Polyp, Reinke’s edema, Papillomatosis and Mild Dysplasia. The malignant group included patients diagnosed with Severe Dysplasia, Carcinoma in situ and SCC.

For further parameter settings and feature evaluation procedure, 80% and 20% of CE-NBI images of the whole dataset were assigned to the training and testing sets, respectively. The images of patients were exclusively tied to separate sets in order to limit the chance of possible over-fitting. Training set was used for hyperparameter optimization as well as training process and testing set was used to evaluate the performance of the features.

### 3.2. Parameter Settings

Following the Equations (1) to (4) and their computation, six parameters needed to be defined. CE-NBI images in the training set were used to find the optimum number of trajectories $N_{k}$ and the length of each section *L*. The values from 50 to 800 with step size of 50 were set to find the $N_{k}$. As the CyEfF values of the selected images did not change significantly for $N_{k}>500$, the number of trajectories $N_{k}$ was set to 500. *L* was defined within the range of 1 to 10 pixels, with the step size was equal to one. The optimum CyEfF values of the selected images were achieved at L=2 pixels. To transform the pixel to the meter unit, we assumed that the longest path in the image is equal to the approximately longest path in Tour de France (200,000 m). The gravitational force *g*, the cyclist-bike weight *m*, and $C_{r}$ related with tires and road characteristics are constant values (g≈9.8 (m/s${}^{2}$), m=80 (Kg) [26], $C_{r}=0.005$ [26]). For this work, the cyclist velocity *v* can also be taken as a constant, and we have set this value to 11 (m/s), which corresponds to the average velocity in the Tour de France.

### 3.3. Feature Evaluation Procedure

The performance of the proposed features was compared with two other feature sets presented in the literature for classifying larynx endoscopic images: Geometrical Features (GF) and Entropy Features (EF).

- The GF set describes the level of disorder of vascular patterns in CE-NBI images [23,24]. This set of features intended to take into account geometrical characteristics of vessels including the consistency of gradient direction and the vessels’ curvature and showed high performances on CE-NBI classification in different datasets [24,25].
- The EF set was used in combination with other types of features for classifying laryngeal tissue in NBI images. We converted each image into a grey-scale level and then divided it into seven different patch sizes of 50×50, 100×100, 150×150, 200×200, 250×250, 300×300 pixels and the whole image. In each patch, the entropy was computed following [21] and stored in a matrix. The mean and variance were computed as features for each image.

GF includes 24 features (F1 to F24), EF 13 Features (F25 to F37) and CyEfF two features (F38 and F39). In order to reduce the very-low frequency trends in the image that can affect the features computation, a homogenization filter was first applied to the image before the features’ computation [24,28].

Two classification scenarios were conducted to evaluate the performance of the feature sets for the classification of CE-NBI images into benign and malignant classes. For that, four supervised classifiers including Support Vector Machine (SVM) with Polykernel and Radial Basis Function (RBF) [29], k-Nearest Neighbours (kNN) [30], and Random Forests (RF) [31] were used. First, each feature set was individually exposed to the classifiers to compare their ability in classifying CE+NBI images. Second, the combinations of feature sets were created by adding EF and CyEfF to the GF. This scenario was performed to see how the proposed features (CyEfF) and the already used features for texture characterization in endoscopy images (EF) can improve the classification performance of the GF.

A 10-fold Cross-Validation with grid search method was used on training data and all feature sets for hyperparameter optimization. Then, the optimized parameters were applied to create the predictive model of classifiers for every feature sets. The features calculated from the CE-NBI images in the testing set with 10-fold Cross-Validation was used to evaluate these predictive models. A confusion matrix was computed in each testing scenario and the accuracy, sensitivity and specificity were obtained from it.

The optimization was conducted to find the value of the regulation parameter (*C*) and kernel parameter(γ) for the SVM classifier. The values within the range of 0.001 to 1000 with a ten-fold increment were assigned for both parameters. The SVM with Polykernel demonstrated the highest performance with C=1 and the SVM with RBF indicated the best results with C=1 and γ=0.01.

For optimizing the kNN performance, the Euclidean distance was used as distance metric. Also, values within the range of 1 to 1000 with step size equal to one were used to select the optimum *k*. The optimum performance of the kNN classifier was obtained at k=10.

The values of depth of trees and the number of estimators were adjusted to reach the optimized performance of RF. The number of estimators were defined within the range of 1 to 1000, with an increase of five. For the depth of the trees, values from 1 to 50 with step size equal to one were set. The classifier showed the highest overall performance at a depth of 7 with 60 trees.

Two feature ranking methods, including *t*-test [32] and Wilcoxon signed-rank test [33], were used to find the top-ranked features that have more influence on the classification results. The *t*-test investigates how significant the differences between groups are. It provides *p*-values as well. A *p*-value is the probability that the results from sample data occurred by chance. In most cases, *p*-value of 0.05 is accepted to mean the data is valid. Wilcoxon signed-rank test can be used to identify if samples from two independent yet related distributions are significantly different.

## 4. Results and Discussion

**Figure 3:**
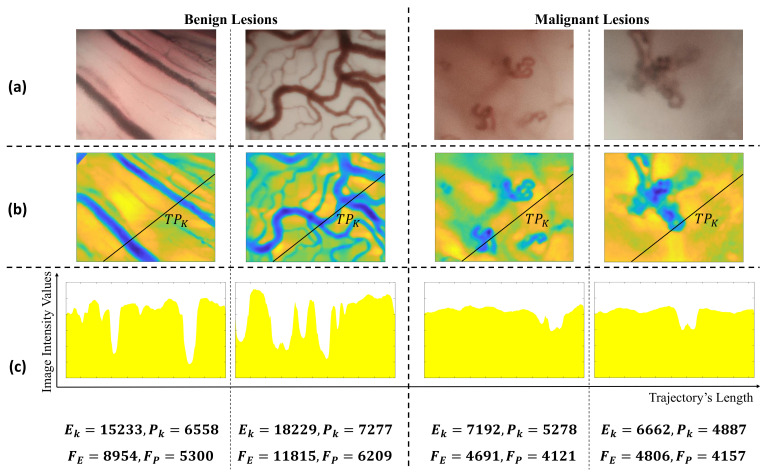
(**a**): Original CE-NBI image, (**b**): Pre-processed image with one random trajectory, (**c**): The stage profile of the random trajectory plus the cyclist’s energy and power values of the random trajectory and the 500 trajectories (whole image).

Figure 3 shows a qualitative example of one trajectory on four different CE-NBI images associated to benign and malignant lesions. Based on the $E_{k}$ and $P_{k}$ values in Figure 3c, the energy and power of the trajectory is significantly different between benign and malignant images. Furthermore, the $F_{E}$ and $F_{P}$ values as the two CyEfF show the variation between two groups of images.

Table 1 shows the classification results of the first scenario described in the previous section. The SVM classification results with Polykernel and RBF had the highest accuracy of 0.882 and 0.875 using CyEfF. With the kNN and RF, GF showed the highest performance with the accuracy of 0.885 and 0.920, respectively. According to the performed result, CyEfF, with only two features, achieved comparable results than GF and EF, which used 24 and 13 features, respectively.

In studies [24,25], a subset of current CE-NBI image dataset were used for classification of CE-NBI images into benign and malignant classes. In comparison to the results in [24], the CyEfF set with Polykernel and RBF SVM showed a better classification accuracy. Furthermore, CyEfF with Polykernel SVM and kNN showed higher sensitivity and specificity than the results presented in [25].

Table 2 presents the results of the second classification scenario in which the combination of GF and CyEfF showed better performance than the combination of GF and EF. The classification accuracy of four classifiers increased from 3 to 12 percent by adding the two proposed features to the 24 GF, in which the highest accuracy of 0.966 was achieved with the kNN classifier. The combination of GF and CyEfF with four classifiers showed higher accuracy, sensitivity and specificity in comparison to the results in [24,25] to classify CE-NBI images into benign and malignant classes. Moreover, in comparison to the other texture-based feature extraction methods such as local binary patterns (LBP) and gray-level co-occurrence matrix (GLCM) that were applied to the laryngeal tissue classification in NBI laryngoscopy [21], the combination of CyEfF and GE feature sets with Polykernel SVM showed the higher performance. These results prove the significant effect of the CyEfF on improving the classification of CE-NBI images with the already used GF set.

**Figure 4:**
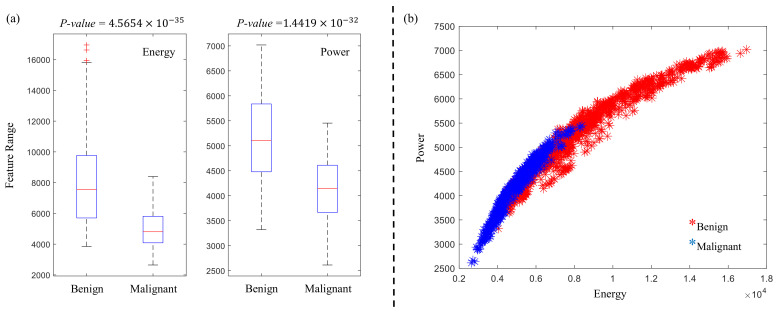
(**a**): Box plot of energy and power features. (**b**): Projected data points of benign and malignant classes using CyEfF.

In order to confirm the significance of the proposed CyEfF, Table 3 shows 10 top-ranked features for each ranking method. Energy (F38) and power (F39) features are ranked as the top 10 features along with some features from GF set in both ranking methods. Figure 4a shows the box plot of energy and power features with the *p*-values equal to $4.5654\times 10^{-35}$ and $1.4419\times 10^{-32}$, computed from the *t*-test, respectively. Based on these values, the proposed features showed a statistically significant difference between benign and malignant classes. Also, Figure 4a shows, that the range of energy and power features for benign and malignant classes are distinguishable. Figure 1b presents that the combination of energy and power features has a separation among benign and malignant classes. These results prove the influence and significance of the proposed CyEfF on the classification results.

The very-low frequency trending characteristics of the image background are usually highly problematic for extracting features since significant pixel values involve progressive changes in the image plane that may affect the extraction of important texture information. In order to study how this type of noise can affect the proposed features, we have computed the method performances by removing the homogenization pre-processing stage. Results show that the classification performance does not significantly vary with or without the pre-processing stage. The accuracy of 0.868, 0.867, 0.850 and 0.872 using SVM with Polykernel and RBF, kNN and RFC were achieved without the pre-processing, respectively. In comparison to the results in Table 1, the accuracy varied only 1 to 2 percent.

## 5. Conclusions

CyEfF approach is an understandable and intuitive method that showed promising results with less amount of data for training in comparison to other deep learning-based feature extraction methods. According to the presented results, CyEfF can describe the textural characterization of CE-NBI images with only two features, which is one of the main advantages of this approach over other hand-crafted feature extraction methods. Moreover, removing the pre-processing stage related to attenuation of very low-frequency trending characteristics of the image did not significantly affect the classification performance of proposed features. However, further evaluation should be conducted for this matter in future work.

As the focus of this paper is on the CE-NBI images, we compared only the performance of the proposed features with other research works in the field of CE-NBI endoscopic imaging modality [23,24,25]. For this reason, comparative experiments to already existing texture-based feature extraction methods on this dataset would be suggested for further development.

Based on the recent advances and improvements in the field of CNN-based approaches, there is a high probability that the application of these methods can result in better in better performance in CE-NBI classification and can overcome the critical limitations of the hand-crafted feature extraction methods. However, it will take a great amount of time to collect and label the data to develop such a method in the medical field for real clinical use. According to our knowledge, there is no CNN-based method for the classification of CE-NBI images in the literature. Hence, the comparison between deep learning-based and hand-crafted feature extraction methods for CE-NBI classification is necessary for future developments.

In spite of the technological advancements, differentiation between malignant and benign lesions in the larynx is difficult in reality, irrespective of the clinicians’ level of experience. In addition, the subjectivity in laryngeal cancer diagnosis has been reported several times, resulting in invasive surgical biopsy and subsequent histological examination. CyEfF in combination with GF as part of a CAD system can potentially solve these problems in CE-NBI image classification and help the clinicians to make final decisions about the stage of laryngeal cancer in the routine and surgical procedures.

With the primary objective of this work to present the significance of the CyEfF for CE-NBI image classification, testing the proposed set of features in other imaging modalities is something that should be accomplished for future work. Based on the presented results, the proposed approach can be used as a new texture-feature extraction method in medical image analysis. For example, it can be applied to the fundus images, as the level of tortuosity of vessels in these images is also crucial for clinicians.

## Figures and Tables

**Table 1:** Classification results of four classifiers using three features sets.

Classifiers	Accuracy			Sensitivity			Specificity		
	GF	EF	CyEfF	GF	EF	CyEfF	GF	EF	CyEfF
SVM with Polykernel	0.820	0.739	0.882	0.818	0.792	0.845	0.822	0.596	0.924
SVM with RBF	0.806	0.761	0.875	0.817	0.802	0.826	0.821	0.515	0.920
kNN	0.885	0.781	0.874	0.911	0.812	0.834	0.836	0.531	0.911
RF	0.920	0.788	0.859	0.935	0.801	0.831	0.892	0.538	0.886

**Table 2:** Classification results of four classifiers using combination of feature sets.

Classifier	Accuracy		Sensitivity		Specificity	
	GF+EF	GF+CyEfF	GF+EF	GF+CyEfF	GF+EF	GF+CyEfF
SVM with Polykernel	0.782	0.944	0.816	0.942	0.738	0.947
SVM with RBF	0.773	0.897	0.813	0.981	0.702	0.818
kNN	0.795	0.966	0.837	0.959	0.718	0.973
RF	0.808	0.956	0.831	0.952	0.724	0.961

**Table 3:** Feature ranking results: F01-F24: GF, F25-F37: EF and F38, F39: CyEfF.

Method	Ranking									
	#1	#2	#3	#4	#5	#6	#7	#8	#9	#10
*t*-test	F38	F21	F39	F14	F24	F22	F09	F20	F17	F15
Wilcoxon signed-rank	F14	F38	F39	F21	F24	F08	F09	F22	F15	F07

## Abbreviations

The following abbreviations are used in this manuscript:

CE	Contact Endoscopy
NBI	Narrow Band Imaging
CyEfF	Cyclist Effort Features
GF	Geometrical Features
EF	Entropy Features
CAD	Computer-Aided Diagnosis
DCNN	Deep Convolutional Neural Network
CNN	Convolution Neural Networks
SCC	Squamous Cell Carcinoma
SVM	Support Vector Machine
RBF	Radial Basis Function
kNN	k-Nearest Neighbours
RF	Random Forests
